# Pituitary Apoplexy Presenting With Oculomotor Nerve Palsy and Headache as the Initial Symptoms: A Case Report

**DOI:** 10.1002/ccr3.70178

**Published:** 2025-02-07

**Authors:** Qifan Hou, Lixin Xu, Jing Yi

**Affiliations:** ^1^ Department of Neurosurgery, Changde Hospital, Xiangya School of Medicine Central South University (The First People's Hospital of Changde City) Changde Hunan China

**Keywords:** case report, headache, oculomotor nerve palsy, pituitary apoplexy

## Abstract

Pituitary apoplexy is a rare clinical syndrome. This report presents a case with initial symptoms of oculomotor nerve palsy and headache. A 48‐year‐old patient reported blurred vision in the right eye for 1 month, followed by a sudden onset of left eyelid ptosis and a 1‐day headache. Laboratory tests revealed normal pituitary function, except for an elevated growth hormone level (> 36.600 μg/L). Preoperative computed tomography (CT) and magnetic resonance imaging (MRI) scans indicated space‐occupying lesion in the sellar region. The lesion was subsequently resected via transnasal subtotal surgery. Histopathological and immunohistochemical analyses confirmed a pituitary adenoma with infarction. The patient received hydrocortisone preoperatively and prednisone and levothyroxine postoperatively. On the second postoperative day, the headache resolved, and the left eyelid regained normal function within 2 weeks. Pituitary apoplexy is extremely rare, with initial presentations of oculomotor nerve palsy being exceptionally uncommon. Early diagnosis and prompt surgical intervention are essential to preserve pituitary function and rapidly alleviate cranial nerve dysfunction.

## 1 Introduction

Pituitary apoplexy is a rare condition, with pituitary adenomas occurring in 3.9 to 7.4 cases per 100,000 people. In the general population, the clinically relevant incidence of pituitary adenomas is about 1 case per 1000 people [1], with 2%–12% of non‐functional adenomas potentially complicated by pituitary apoplexy [2]. Cases of pituitary apoplexy presenting with oculomotor nerve palsy as the initial symptom are even rarer. This article presents a case of pituitary apoplexy and discusses its diagnosis and management.

## 2 Case Report

**FIGURE 1:**
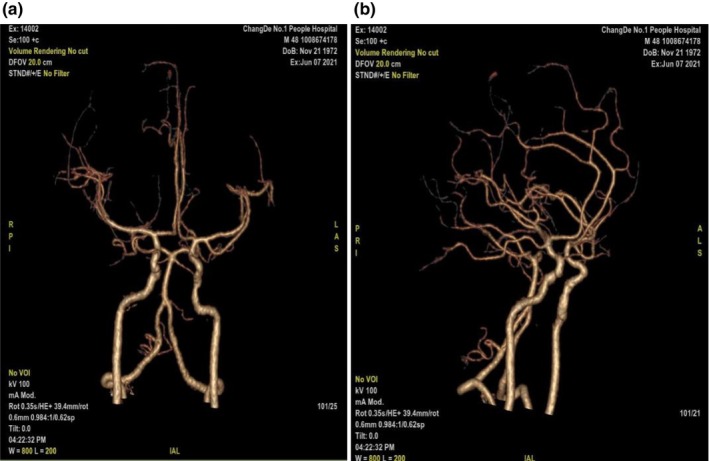
Cerebral CT angiography reveals: No obvious intracranial aneurysm or vascular malformation was observed.

**FIGURE 2:**
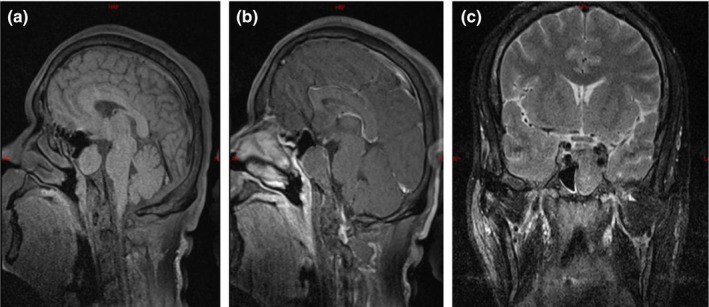
MRI of the pituitary gland with plain and enhanced scans: An irregularly shaped, heterogeneously slightly T1 and T2 hyper signal intensity is present in the sphenoid sinus, with partial absorption of the overlying bone. The mass protrudes into the intracranial space, and slight mild enhancement is observed upon enhancement. The pituitary gland is compressed and slightly deviated to the right.

A 48‐year‐old male patient presented with 1 month of blurred vision in the right eye and a sudden onset of left‐sided headache and left eyelid ptosis beginning 1 day ago. One month ago, the patient was diagnosed in our ophthalmology department with right eye vitreous hemorrhage, bilateral diabetic retinopathy, bilateral cataracts, and type 2 diabetes. The cause of blurred vision remained unclear. One day, the patient experienced a sudden left‐sided headache, left eyelid ptosis, and limited movement of the left eyeball inwards, upwards, and downwards. This patient was diagnosed with diabetes before admission and regularly received insulin for hypoglycemic treatment. He has smoked for more than 30 years, 330 cigarettes a year, but has no obvious family medical history. The patient was conscious and presented with left eyelid ptosis; left eye movement was restricted. The left pupil was round with a diameter of approximately 3.5 mm and lacks a light reflex, while the right pupil was round with a diameter of approximately 2 mm and had a sluggish light reflex. Initial visual acuity in the left eye was 0.4, with no field defects. Pituitary function tests show elevated growth hormone levels, with all other values within normal limits (Table 1). Cerebral CT angiography revealed no obvious intracranial aneurysms or vascular malformations (Figure 1). MRI of the pituitary gland, both plain and enhanced, revealed an irregularly shaped mass with slightly T1 and T2 hyper signal intensity in the sphenoid sinus, with partial absorption of the overlying bone. The mass protruded into the intracranial space, with mild enhancement observed on imaging. The pituitary gland was compressed and deviated to the right (Figure 2).

**TABLE 1:** Basic preoperative and postoperative endocrinology evaluation.

	Measurement level, preoperative	Measurement level, postoperative	Normal level
TSH	2.047 μIU/mL	0.217 μIU/mL↓	0.56–5.91 uIU/mL
T3	1.35 pmol/L	0.630 pmol/L ↓	0.92–2.38 nmol/L
T4	149.4 pmol/L	107.680 pmol/L	69.71–163.95 nmol/L
FSH	10.41 mIU/mL	4.29 mIU/mL	1.4–18.1mIU/mL
LH	3.94 mIU/mL	1.13 mIU/mL↓	1.5–9.3 mIU/mL
PRL	5.86 ng/mL	0.85 ng/mL↓	2.1–17.7 ng/mL
HGH	> 36.600 μg/L	0.413 μg/L	0.003–0.971 μg/L
TESTO	4.50 nmol/L	0.55 nmol/L↓	4.27–28.24 nmol/L

Abbreviations: FSH, follicle stimulating hormone; HGH, human growth hormone; T4, thyroxine.

## 3 Diagnosis and Results

**FIGURE 3:**
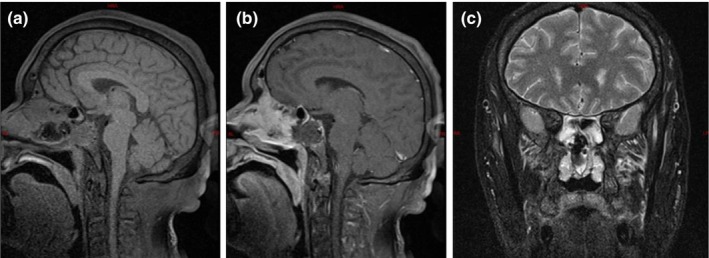
Postoperative reexamination of pituitary MRI shows normal morphology and uniform internal signal. No obvious abnormal enhancement area is observed. The shape of the pituitary stalk is normal and centered. There are irregular mixed signals in the left sphenoid sinus, with enhanced areas showing enhancement.

**FIGURE 4:**
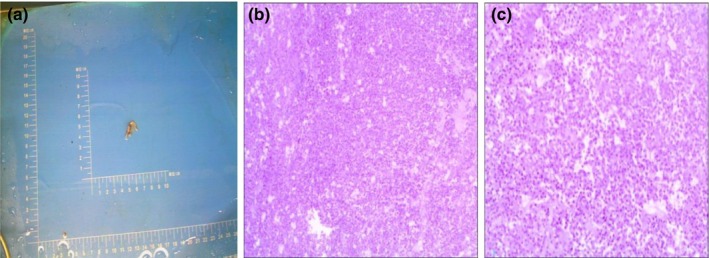
Pathology results: The presence of an infarct within a pituitary adenoma was observed.

Based on the findings, the initial diagnosis suggests a mass lesion in the sphenoid sinus, with the nature still undetermined. Before surgery, the patient was administered 150 mg of oral hydrocortisone. An endoscopic transnasal transsphenoidal approach was then used to resect the pituitary lesion in the sella region. The excised tumor tissue was sent for histopathological and immunohistochemical analysis after the procedure. Intraoperatively, the tumor appeared soft, with a cottage cheese–like consistency and a yellow‐white hue, showing minimal vascularity. A week post operation, a follow‐up assessment of the pituitary hormone levels indicated a reduction in thyroid‐stimulating hormone (TSH), triiodothyronine (T3), luteinizing hormone (LH), prolactin (PRL), and testosterone (TESTO) levels. Given the patient's surgical history and hormonal deficiencies, prednisone and levothyroxine were prescribed to manage pituitary insufficiency. Postoperative reexamination of pituitary MRI shows normal morphology and uniform internal signal. No obvious abnormal enhancement area is observed. The shape of the pituitary stalk is normal and centered. There are irregular mixed signals in the left sphenoid sinus, with enhanced areas showing enhancement (Figure 3). The tumor specimen revealed a pituitary adenoma with infarction (Figure 4). Immunohistochemistry results: GFAP (−), GH (+), CD56 (+), Syn (+), CgA (+), PRL (+), TSH (−), ACTH (−), FSH (−), LH (−) and Ki67 (+, 1%). The patient reported headache relief by the second postoperative day. One week after surgery, the left eyelid ptosis improved, allowing partial elevation. The left pupil diameter measured approximately 3.5 mm, and both direct and indirect light reflexes were absent. Additionally, the left eyeball was unable to move upwards, downwards, or towards the nasal side. Full recovery of the deficit was at 2 weeks after the surgery.

## 4 Discussion

Pituitary apoplexy (PA) is a rare clinical syndrome [2, 3, 4]. It is typically caused by hemorrhage or infarction of a preexisting pituitary adenoma. Since the primary event involves the adenoma, the syndrome should be referred to as pituitary tumor stroke, rather than pituitary apoplexy [3].

PA is more common in men aged 50–60 years and is rare in children and adolescents [5]. The incidence of pituitary adenomas is 3.9–7.4 per 10,000 individuals, and clinically relevant pituitary adenomas occur in about 1 in 1000 cases in the general population [1]. Between 2% and 12% of pituitary adenomas, particularly non‐functional tumors, may develop PA. The main symptom is sudden severe headache, sometimes accompanied by visual impairment or ophthalmoplegia, and sometimes combined with partial or complete endocrine dysfunction [2, 6, 7]. Helen et al. described a patient with hemorrhagic infarction of a pituitary adenoma, presenting with blurred vision due to oculoparesis shortly after hip replacement, and hyponatremia caused by cortisol deficiency. Accurate assessment of the etiology and appropriate treatment are essential [8].

In a retrospective study of 12 patients with pituitary tumors combined with cranial nerve palsy, Sang Hyun Kim et al. analyzed their clinical features, imaging findings, and postoperative effects. They found that the third cranial nerve was most frequently affected, followed by the sixth and fourth cranial nerves. These symptoms were recovered in the opposite order. Recovery time after cranial nerve palsy was strongly correlated with the interval between symptom onset and surgery [9]. Additionally, a 45‐year‐old diabetic male from Ethiopia was reported to experience severe hypoglycemia caused by acute hypopituitarism secondary to PA. Hypoglycemia commonly occurs in diabetic patients on sulphonylurea monotherapy. However, when severe enough to alter mental status, hypoglycemia requires distinct treatment approaches. Clinical signs of cortisol deficiency may indicate hypopituitarism as the underlying cause [10]. In contrast to hypoglycemia, Mannmohan K. Kamboj et al. reported a rare case of PA in an 18‐year‐old male with no prior diagnosis of type 2 diabetes, presenting with unexplained hyperglycemia (49.2 mmol/L [887 mg/dL]) and obtundation [11].

Recognized predisposing factors for PA include increased intracranial pressure, arterial hypertension, major surgery, anticoagulation therapy, or dynamic testing [2]. Additionally, head trauma and pregnancy are also potential triggers for PA [12]. A 68‐year‐old male presented to the emergency department with head trauma from a fall, reporting severe headache, nausea, vomiting, and diplopia. MRI revealed a giant pituitary adenoma with intratumoral hemorrhage and acute tumor expansion, compressing the abducens nerve laterally. Isolated ocular abducens nerve palsy caused by posttraumatic PA is rare and often clinically diagnosed due to the nonspecific nature of its symptoms and signs [6]. Frequent ischemic infarction in pituitary adenomas arises from their intrinsic characteristics. These intrinsic properties create a delicate balance between high metabolic demands and limited tissue perfusion. Consequently, these tumors are prone to spontaneous infarction or acute ischemic events caused by factors such as systemic hypotension, reduced nutrient supply, hypoglycemia, or increased metabolic demands from hypothalamic regulation.

These intrinsic features of pituitary adenomas could be leveraged to exploit their vulnerabilities and develop novel therapeutic strategies [13]. The cisternal segments of the oculomotor nerve typically course through the medial part of the oculomotor triangle before entering the oculomotor cistern. Oculomotor nerve paresis can result from vascular compression at any location within the interpeduncular cistern or oculomotor triangle [14].

Following PA, a sudden increase in the contents of the saddle region compresses the surrounding structures and portal [15, 16]. Hiroyuki Kobayashi et al. described two cases of PA without cavernous sinus invasion, presenting with isolated oculomotor palsy. By analyzing CT and MRI, they speculated that oculomotor palsy could first be caused by unilateral erosion of the posterior clinoid process, leading to lateroposterior protrusion of the adenoma. Hemorrhage may cause sudden kinking of the oculomotor nerve at the entrance of the oculomotor trigone [17]. The superolateral and posterior components of the cavernous sinus represent potential pathways for PA invasion. Improved identification of the cavernous sinus breakthrough pattern is crucial for achieving higher total resection and remission rates [18].

CT or MRI can generally reveal hemorrhagic or necrotizing pituitary tumors, thereby confirming the diagnosis of PA [2]. MRI is the preferred imaging modality [13]. Regardless of vascular risk factors, brain MRI and laboratory tests play a critical role in the initial evaluation of elderly patients with isolated acute oculomotor nerve palsy [19]. PA was previously considered a neurosurgical emergency requiring surgical intervention [2]. Treatment now varies based on clinical presentation, though urgent or emergent surgical resection is often required [7]. The decision to pursue a conservative approach in a stable patient with pituitary apoplexy resulted in quick resolution of a third nerve palsy in our patient. This had the advantage of avoiding the need for surgical resources and potential complications that may arise with surgery [20].

The prognosis is excellent for most PA patients. Patients selected can be managed conservatively and patients with severe neuronal ophthalmological defects can achieve good recovery with early surgical treatment [21]. If conservative treatment is chosen, close clinical monitoring is necessary for early detection of deterioration. Most patients will have residual hormonal deficiencies, which will require long‐term hormone replacement therapy. Patients with residual tumor have a small but significant risk of PA recurrence, especially in patients with large tumor residues. The risk of tumor recurrence after PA is also small, so all patients should be monitored regularly by imaging to detect possible tumor recurrence [12]. The time required for recovery after cranial nerve palsy was clearly correlated with the length of time between symptom onset and surgery. Early surgical intervention is most likely to allow a rapid recovery of cranial nerve dysfunction [9]. Overall, although PA is an uncommon complication of pituitary tumors, timely diagnosis and treatment are needed [15].

## 5 Conclusion

Pituitary apoplexy is a rare clinical syndrome, with cases where oculomotor nerve palsy as the initial symptom being exceptionally uncommon. A review of the literature suggests that for individuals suspected of pituitary apoplexy, prompt laboratory tests, MRI, CTA, and further diagnostic evaluations are essential to confirm the diagnosis and enable early surgical intervention. This approach aims to preserve the pituitary function and address any cranial nerve impairments.

## Author Contributions

**Qifan Hou:** data curation. **Lixin Xu:** investigation, resources. **Jing Yi:** writing – review and editing.

## Ethics Statement

This research was approved by the ethics committee of Changde Hospital, Xiangya School of Medicine, Central South University.

## Consent

Written informed consent was obtained from the patient to publish this report in accordance with the journal's patient consent policy.

## Conflicts of Interest

The authors declare no conflicts of interest.

## Data Availability

The data used to support the findings of this study have been included in this article.
